# Human and entomological determinants of malaria transmission in the Lihir Islands of Papua New Guinea: A cross-sectional study

**DOI:** 10.1371/journal.pntd.0012277

**Published:** 2025-01-03

**Authors:** Pere Millat-Martínez, Michelle Katusele, Bernadine Kasian, Elias Omera, Esther Jamea, Lina Lorry, Aina Casellas, Dan Ouchi, Chilaka Wali, Sylvia Raulo, Arthur Elizah, Peter Kaman, Absalom Dau, Muker Sakur, Lemen Kilepak, Siub Yabu, Nelson Koata, John Kave, Michael Toa, Christopher Urakusie, Charles Kongs, Frank Kisba, Moses Laman, Oriol Mitjà, William Pomat, Quique Bassat, Stephan Karl, Bàrbara Baro

**Affiliations:** 1 ISGlobal, Barcelona, Spain; 2 Papua New Guinea Institute of Medical Research, Goroka, Eastern Highlands Province, Papua New Guinea; 3 Lihir Malaria Elimination Programme, Lihir Island, New Ireland Province, Papua New Guinea; 4 Fight Infectious Diseases Foundation, Hospital Germans Trias i Pujol, Badalona, Spain; 5 School of Medicine and Health Sciences, University of Papua New Guinea, Port Moresby, National Capital District, Papua New Guinea; 6 Centre for Health and Social Care Research (CESS), Faculty of Medicine, University of Vic—Central University of Catalonia (UVic—UCC), Vic, Catalonia, Spain; 7 Lihir Medical Centre, International SOS, Lihir Island, New Ireland Province, Papua New Guinea; 8 Facultat de Medicina i Ciències de la Salut, Universitat de Barcelona (UB), Barcelona, Spain; 9 ICREA, Pg. Lluís Companys 23, Barcelona, Spain; 10 Paediatrics Department, Hospital Sant Joan de Déu, Universitat de Barcelona, Esplugues, Barcelona, Spain; 11 Centro de Investigação em Saúde de Manhiça (CISM), Maputo, Mozambique; 12 CIBER de Epidemiología y Salud Pública, Instituto de Salud Carlos III, Madrid, Spain; 13 Australian Institute of Tropical Health and Medicine, James Cook University, Cairns, Australia; National Institutes of Health, UNITED STATES OF AMERICA

## Abstract

**Background:**

The Lihir Islands of Papua New Guinea, located in an area with high burden of malaria and hosting a large mining operation, offer a unique opportunity to study transmission. There, we investigated human and vector factors influencing malaria transmission.

**Methods:**

In 2019, a cross-sectional study was conducted on 2,914 individuals assessing malaria prevalence through rapid diagnostic tests (RDT), microscopy, and quantitative PCR (qPCR). A logistic regression analysis identified infection-associated factors. *Anopheles* species distribution, biting behaviours, and sporozoite carriage were assessed through human landing catches and larval surveys.

**Results:**

Overall malaria prevalence (any species) was 3.6% by RDT, 4.5% by microscopy, and 15.0% by qPCR. *P*. *vivax* accounted for 37.1% of infections, *P*. *falciparum* for 34.6%, *P*. *malariae* for 3.0%, *P*. *ovale* 0.2%, and mixed infections for 24.5%. Prevalence (qPCR) varied across geographic areas, from 8.5% in the mine-impacted zone (MIZ) to 27.0% in the non-MIZ. Other factors independently associated with infection risk included cohabiting with an infected individual (aOR = 1.94, 95%CI: 1.56–2.42), and residing in traditional housing (aOR = 1.65, 95%CI: 1.21–2.25). Children had double the infection risk compared to adults, and the use of long-lasting insecticidal-treated nets did not decrease risk of infection. *An*. *punctulatus* was the major vector in one of the four geographical areas; while *An*. *farauti* was predominant in the rest of them, both with an early biting behaviour but with different biting intensities by geographical area. Entomological inoculation rates ranged from 26.9 (95%CI: 12.3–45.2) infective bites per person-year in the MIZ to 441.3 (95%CI: 315.7–572.1) in the non-MIZ.

**Conclusions:**

Malaria transmission and infection was lower in the MIZ compared to other areas. Measures focusing on at-risk groups, including vector-control and transmission interruption methods, could be taken into account by the mine and the healthcare authorities to reduce malaria burden outside the MIZ.

## Author summary

The Lihir Islands of Papua New Guinea host a mining operation on Aniolam, the main island, which implements specific vector control strategies in the mine-impacted zone (MIZ); whereas the remaining areas rely solely on distribution of long-lasting insecticidal-treated nets (LLIN). Our study compared transmission dynamics in the different geographic areas and demonstrated lower malaria incidence, prevalence, *Anopheles* density and biting intensities in the MIZ. Living outside the MIZ on Aniolam and on Malie Island was associated with a higher risk of infection, with transmission metrics rarely seen outside high burden countries in sub-Saharan Africa. Besides the geographic area, other risk factors for infection included younger age, cohabiting with infected individuals, and living in traditional housing; while the use of LLINs was not associated with a reduction of infection risk.

Our results highlight the need for tailored strategies based on local transmission patterns and could be beneficial for ongoing initiatives aimed at malaria control and elimination. In Lihir, there is an opportunity to invest and improve the collaboration between the mine company and public health authorities. Such a public-private partnership could enhance control efforts, including expansion of diagnostics and treatments, the vector control program and community education beyond the MIZ.

## Introduction

Progress in reducing the global malaria burden has stagnated since 2015, and the milestones proposed by the World Health Organization (WHO) have not been met [1]. Lack of progress on malaria control has been shown in the WHO Western Pacific region, where estimates for incidence have increased by 10% and mortality rates by 4% between 2015 and 2021 [2]. This is largely due to a rise in the malaria burden in Papua New Guinea (PNG), as it accounts for 87% of all malaria cases and 94% of all malaria deaths in the region [3]. It is estimated that 35.7% of the PNG population (approximately 3.33 million inhabitants) live in areas with high to moderate malaria risk [4]. Malaria transmission in PNG exhibits geographical heterogeneity, with the northern coast and the islands regions being affected with high levels rarely found outside of Sub-Saharan Africa [5]. *Plasmodium falciparum* and *P*. *vivax* are highly endemic in PNG, although the other two human parasite species, *P*. *malariae* and *P*. *ovale*, are also present [6]. Hence, improving funding for and focusing malaria control efforts in PNG is warranted for reducing malaria transmission in the region [3].

While malaria symptomatic cases can be confirmed by microscopy or rapid diagnostic test (RDT), molecular tools such as polymerase-chain reaction (PCR) are required to confidently detect sub-clinical infections, given many infections present with sub-microscopic parasite densities [7]. Although malaria transmission has been mainly related with presence of gametocytes; these sub-microscopic infections may constitute a source of ongoing transmission, and are a common feature in malaria endemic areas involving all *Plasmodium* species and individuals of all ages [8]. In PNG, the 2019/2020 malaria indicator surveys showed that prevalence of microscopic parasitaemia in household surveys varied among different geographic areas, ranging from 0.1% in the highlands areas to 10.6% in the coastal areas of the country [9]. When PCR is used to include sub-microscopic infections, observed prevalence of malaria parasites increases, as shown in a systematic review for *P*. *falciparum* infections [10]. In *P*. *vivax* endemic settings, this difference between microscopy and PCR can be even higher. As an example, in a cross-sectional study performed in Madang Province during 2014, *P*. *falciparum* prevalence increased from 2.8% to 9.0% when comparing microscopy to PCR results, while *P*. *vivax* prevalence increased from 2.7% to 19.7% [11].

The main mosquito species transmitting malaria in PNG are members of the *Anopheles punctulatus* complex, namely *Anopheles farauti s*.*s*., *An*. *punctulatus s*.*s*. and *An*. *koliensis s*.*s* [12,13], and cryptic species within this complex exist that can be hard to morphologically identify [14]. Significant variation in vector abundance and infective biting rates are observed between villages, even within the same region [15]. While there is no evidence of widespread pyrethroid resistance in the Anopheles populations in PNG [16], some studies suggest behavioural adaptation after the first nationwide distribution of long-lasting insecticidal treated-nets (LLINs) between 2005 and 2009, resulting in a moderate shift towards an earlier peak biting in *An*. *punctulatus* and *An*. *farauti* [17]. A survey conducted in 2016, confirmed that 25.5–50.8% of the vectors in the studied villages encountered human hosts in the evening, with an increased proportion of anophelines feeding outdoors [15].

The Lihir Islands, present unique characteristics to study malaria transmission and evaluate interventions. Aside of being located in one of the provinces with highest malaria transmission in PNG (>200 yearly cases per 1,000 inhabitants) [5], they host the world’s fifth largest gold mining operation on their biggest island, Aniolam. Newcrest Mining Ltd, the mine company, provides essential services to communities within the mine impacted zone (MIZ) surrounding the open-pit (including health services, electric power and waste management systems in all villages of the MIZ, and water supply for some of the villages). In addition, a vector control program has been deployed, which includes regular larval source management in the MIZ and fogging targeting the mining camp areas. As such, since 2006, the company conducts drainage of small water bodies, and larviciding of big water bodies using *Bacillus thuringiensis israelensis* within this area. A prevalence study conducted in 2010, showed a marked reduction in malaria positive children found by microscopy in the MIZ (from 31.5% in 2006, to 5.8% in 2010) [18]. In contrast, the rest of the Lihir Islands rely only on case management provided by the government health facilities and the services provided by the national malaria control program, which includes mass distribution of LLINs every 3 years to achieve universal coverage. Nevertheless, despite achieving 97%-98% coverage (ownership of at least one LLIN for every two people) during the distribution campaigns, only 8.7% of households maintained at least one LLIN for every two individuals two years after distribution [19].

Nearly a decade later, in 2019, we conducted a study to characterize transmission, obtaining incidence data and performing prevalence and entomological surveys in the MIZ, and importantly, in the other geographic areas of the Lihir Islands. Factors associated with high malaria transmission, both in the human and the vector population, were assessed.

## Methods

### Ethics statement

This study was approved by the PNG Medical Research Advisory Committee (PNG-MRAC) with MRAC No.18.07. The informed consent process followed community and cultural values of PNG. Following consultation with and approval by community leaders, awareness meetings or notes (*tok saves*) were delivered in each village to explain the study. Permission for collecting malaria incidence data registered at health facilities was obtained from the health department of the local level government and from the district level government (Namatanai District, New Ireland Province, PNG). For the cross-sectional study, individual written informed consent was obtained from all participants, or the parent or legal guardian of children below 18 years old, after explanation of the risks and potential benefits of the study. Children under the age of 18 were verbally assented. Those participants unable to read and/or write were verbally consented with a witness countersigning the consent form. For the human landing catches, individual written informed consent was obtained from all participants after explanation of the risks and potential benefits of the study, and chemoprophylaxis was offered to prevent infection from exposure to infectious mosquito bites.

### Study setting

This study was conducted in the Lihir Islands, which are located 900 km northeast of Port Moresby and include Aniolam, Malie, Masahet and Mahur islands. A map of the Lihir Islands can be seen in S1 Fig. The population number in the Lihir Islands is estimated to be between 26,500 and 26,800 inhabitants. The villages of the MIZ accommodate half of the Islands’ population, and there are 2,000–3,000 extra mobile workers staying at the mine housing facilities in Londolovit, in the MIZ. Most of the people staying in the MIZ are migrants from other places of PNG (57% of all inhabitants), living in permanent or makeshift houses and less in traditional houses that are more common in other areas of Lihir.

### Collection of health system data

The health system in Lihir is highly fragmented. The MIZ contains the Lihir Medical Centre, run by an external health service provider contracted by the mine company. In contrast, in the non-MIZ, there are public health facilities, including a health centre, a health sub-centre and eight aid posts (S1 Fig), which are under-staffed with community health workers and some nursing officers. Passive case detection data registered between January and December of 2019 in all the health facilities (public and private) were extracted and digitalized for analysis of malaria incidence.

### Study population, data and sample collection

We conducted a cross-sectional study during October and November of 2019. We included all the administrative villages in the Lihir Islands. Random households were sampled from every village following a probability-proportional to size strategy. In each village, all households were enumerated and geo-positioned. The eligible individuals were those living in the randomly selected households (i.e. non-visitor individuals staying in that household for at least the preceding 2 weeks) and present at the moment of the survey. The participants were recruited, stratified by age groups, until the achievement of the sample size objectives (see Statistical analysis section). The number of participants recruited for each village were proportional to the village population size according to the last estimates.

Following informed consent, demographic and clinical data, relation with head of the household, LLIN usage and mobility information were collected from all participants using Open Data Kit. Information on type of house was also collected, which could be permanent (built of bricks, with solid material in the roof and windows with glass), traditional (built of natural materials, especially wood and grass, with open windows) or makeshift (made with different pieces of materials such as metal, cardboard, uralite, stones or wood). Pregnancy was assessed through asking the pregnancy status to all female participants aged ≥ 16 to < 50 years old; no confirmatory test was conducted. A finger prick was performed by a health practitioner, and blood drops were collected for a malaria RDT (Malaria Pf/PAN Ag Combo RDT, Carestart, USA), a blood slide with thin and thick smears for microscopy examination, 2 dry blood spots in filter paper, and a haemoglobin analysis with Hemocue HB 301 analyser. A short clinical assessment, including axillary temperature measurement, spleen size assessment and history of last malaria episode, was conducted by a clinician, who also interpreted the result of the RDT. In case of a positive RDT result, antimalarial treatment with artemether/lumefantrine (plus full unsupervised course of primaquine if *P*. *vivax* was detected) was delivered following doses recommended by PNG guidelines [20]. In case of anaemia, the participants were referred to the nearest health facility for assessment.

The laboratory procedures for the analysis of samples using light microscopy and quantitative PCR (qPCR) were standard and are described in the Supplementary methods in S1 File.

### Mosquito and larval sampling

In parallel to the cross-sectional study, we conducted an entomological survey including human landing catches (HLC) and larval collection at eight sentinel sites distributed throughout Lihir: 3 villages in Aniolam MIZ, 3 villages in Aniolam non-MIZ, 1 village in Malie and 1 village in Masahet. These sites were selected after studying the environmental characteristics of Lihir Islands to represent the ecological diversity found within Aniolam, as well as in the outer Islands, and to evenly cover locations within and outside of the MIZ. For the HLC, 20–25 healthy consented volunteers were selected in each site. The inclusion criteria were those individuals, male or female, from 18 to 75 years old living in the selected site, and expressing a willingness to participate in HLC. Exclusion criteria were those participants who were not willing to give informed consent, and individuals with any apparent acute or chronic illness. The procedures for the HLC and the analyses of the mosquito biting frequency, biting time, indoor vs. outdoor biting, and the entomological inoculation rate (EIR) are described in the Supplementary methods, in S1 File.

All potential larval habitats that could be found around at the eight collection sites were surveyed and categorized as confirmed or potential larval habitat depending on the presence of *Anopheles spp*. larvae. Two more sites for potential larval habitats were included (one in Aniolam-MIZ and one in Aniolam non-MIZ). Density per habitat was estimated using a larval dipping method, and GPS coordinates and environmental variables were recorded. This allowed for mapping and characterizing each habitat. All larvae were reared to adults in a temporary field insectary, and were identified to species using light microscopy.

The methods used for the molecular identification of Anopheles species and *Plasmodium* sporozoites in the adult mosquitoes’ samples are described in detail in the Supplementary methods, in the S1 File.

### Statistical analysis

The sample size for the estimation of malaria prevalence in the general population was calculated for a precision of 1.5% and a confidence interval (CI) of 95%, with an estimated malaria prevalence of 20% based on the 2010 data [18], yielding a minimum required population to screen of 2734 participants. Specific sample sizes for each village were determined according to the described probability-proportional to size strategy.

Data were described as absolute and relative frequencies for categorical variables. For continuous variables we described mean and standard deviation (SD) for normally distributed data and median and interquartile range (IQR) for skewed data. Chi-squared tests (or Fisher’s exact tests) and t-tests were performed to assess differences between groups for categorical and quantitative variables, respectively. Spearman’s Rank correlations were calculated to estimate the relationship between quantitative variables. Univariable and multivariable logistic regression models were used to determine the factors that were associated with PCR positivity. For the incidence, a binomial regression model was fitted to estimate the incidence risk ratio (IRR) and the Wald 95% CI; and the Mann-Kendall statistical test was employed for trend analysis to consider for seasonal patterns present in monthly incidence time series.

Mosquito collections conducted over the course of one night were considered to be equivalent to one person-night. It was assumed that the number of anopheline mosquitoes landing on a collector was equivalent to the number of mosquito bites. Biting frequency distributions over the course of the night were graphed as the mean frequency of mosquitoes sampled hourly from 6 pm to 6 am. Sporozoite rate was quantified as the proportion of PCR-tested mosquitoes that were positive for malaria parasites. Variation of sporozoite rates was assessed using Chi-squared test. Human biting rate (HBR) was quantified determining the total number of mosquitoes collected divided by the number of nights of collection and the number of collectors (i.e., the total exposure time). It was multiplied by 365.25 to obtain the HBR per person-year. 95% CI of each rate was calculated using the Wald method. EIR was estimated by multiplying the HBR and the sporozoite rate. It should be noted that the EIR calculated in the present study is not reflective of an annual average transmission intensity. It should be considered as a relative estimate of transmission intensity at the time of measurement. Variation in vector composition and metrics among sampling location was tested using Chi-squared test.

A correlation analysis was performed taking into account the HBR and EIR, and the results of the prevalence and the incidence of the next month after mosquito collection, in each of the eight villages with mosquito collection. Spearman’s Rank was used for the calculation of the correlations between the human and vector data, and 95% CI and p-values were reported.

The computer packages STATA and R were used for the analysis of epidemiological and entomological data [21,22]. The significance level of all statistical tests was based on type I error rate of 0.05.

## Results

### Study population

A total of 2,914 individuals of all ages above 6 months and residing in 696 households across the 42 villages of Lihir Islands were included in the cross-sectional study. Demographic, geographic and clinical characteristics of participants are summarized in S1 Table. Sex and age of the participants were representative of the Lihir Islands population, with 54.5% females and a predominance of younger individuals: <5 years, 12.4%; 5–14 years, 20.8%; 15–24 years, 21.1%; 25–34 years, 20.4%; 35–44 years, 11.4%; ≥45 years, 13.9%. Half of the participants (49.2%) were living in the MIZ of Aniolam, 33.5% in the rest of Aniolam (non-MIZ), 8.3% in Masahet Island, 4.9% in Mahur Island, and 4.0% in Malie Island. The type of house differed across Aniolam, with less traditional houses in the MIZ compared to the non-MIZ (9.3% vs 17.7%, p<0.001; Chi-squared test).

Of all participants, 649 (22.3%) had a history of fever in the preceding month, but only 23 (0.8%) had a documented axillary body temperature ≥37.8°C, and 46 (1.6%) were taking an antimalarial at recruitment. Haemoglobin levels were below 12 g/dL in 70.4% of the participants and increased with age, with a mean of 9.4 g/dL in under 5 years-old, 10.5 g/dL in 5–14 years-old, and >11.0 g/dL in all groups older than 15 years-old (p<0.0001; Chi-squared test). Haemoglobin <8.0 g/dL was detected in 6.8% of the individuals. Pregnant women had lower haemoglobin levels than their non-pregnant child-bearing age counterparts (9.6 g/dL vs 10.6 g/dL, p = 0.0005; t-test). On the other hand, 7.6% (39/516) of individuals from the population aged ≤ 15 years-old had splenomegaly.

Finally, 37.2% of the participants reported having slept under a LLIN the previous night. LLIN use was more common among females compared to males (41.3% vs 32.3%, p<0.0001; Chi-squared test), while sleeping outdoors was more frequent in males compared to females (6.7% vs 2.9%, p<0.0001). LLIN use also differed across age (p<0.0001), with children under 5 years-old being the ones using LLIN the most (49.3%). When asked on prevention measures to avoid acquiring malaria, less than half of the population (39.6%) responded that sleeping under LLIN prevents malaria.

### Malaria prevalence in the Lihir Islands

Overall, 105 participants (3.6%; 95% CI: 2.9%-4.3%) had a positive malaria RDT, 132 (4.5%; 95% CI: 3.8%-5.3%) had blood-stage parasites of any *Plasmodium* species detectable by light microscopy, and 437 (15.0%; 95% CI: 13.7%-16.3%) had a *Plasmodium* positive qPCR.

RDT showed in 91 (86.7%) participants a single line for histidine-rich protein 2 (HRP2), indicating *P*. *falciparum* mono-infection; in 14 (13.3%) participants it showed a single line for *Plasmodium* lactate dehydrogenase (pLDH), indicating non-*P*. *falciparum* species infection (mainly *P*. *vivax* in this setting); and there were no RDTs with both lines positive (indicating *P*. *falciparum* or mixed infection). In contrast, microscopy showed 67 (50.8%) cases of *P*. *falciparum* infection, 51 (38.6%) of *P*. *vivax*, 7 (5.3%) of *P*. *malariae*, and 7 (5.3%) mixed infections (*P*. *falciparum* and *P*. *vivax)*. No *P*. *ovale* infection was detected by microscopy. Gametocytes were detected in 23 (31.1%) of all positive *P*. *falciparum* samples, with a median (IQR) concentration of 164 (45–1,854) parasites/μL; and in 12 (20.7%) of all positive *P*. *vivax* samples, at 70 (28–229) parasites/μL. Gametocyte prevalence considering both species was 1.2% of the overall study population. On the other hand, median asexual parasitaemia was 442 (147–5,313) parasites/μL in any *P*. *falciparum* positive sample, and 164 (78–853) parasites/μL in any *P*. *vivax* positive sample. Aiming to compare with a previous prevalence study performed in Aniolam (18), we quantified the positive cases found by microscopy in children under 5 years-old, which were 5.0% in the MIZ of Aniolam and 19.9% in the non-MIZ (p <0.0001).

**Fig 1:**
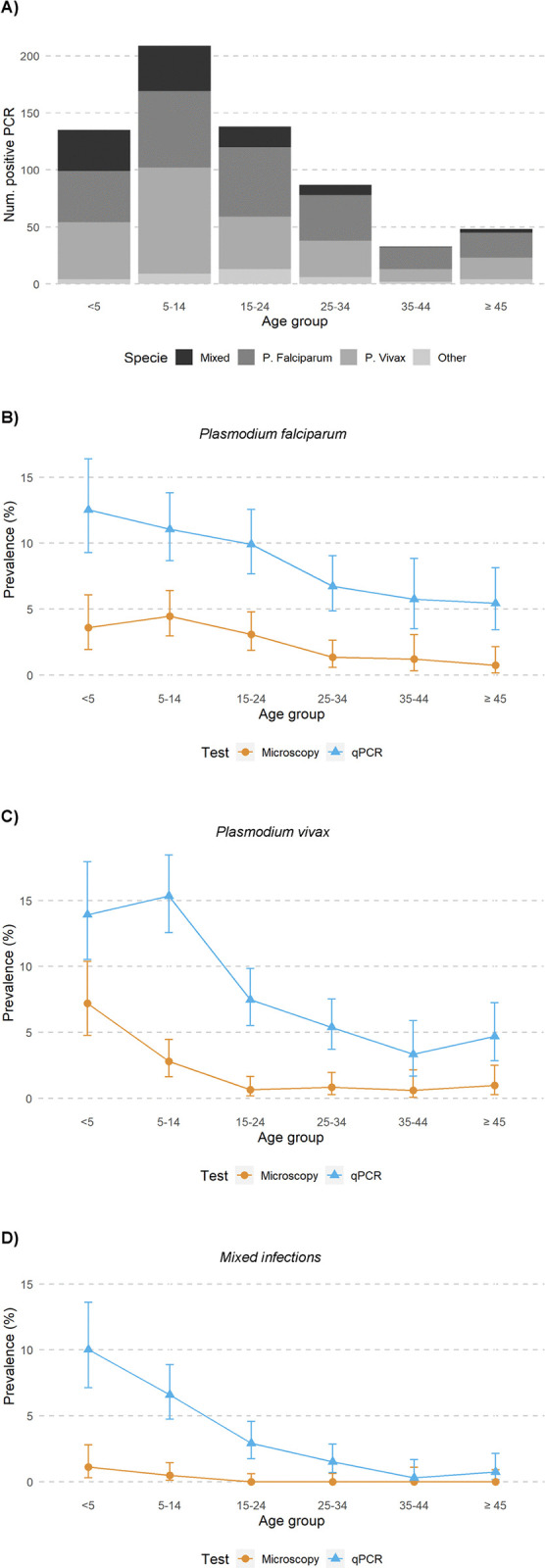
*Plasmodium* species detected in the cross-sectional survey. (A) Number of *Plasmodium* infections detected by qPCR classified by age group and species; (B) Prevalence of *P*. *falciparum* infections by age groups (light microscopy and qPCR); (C) Prevalence of *P*. *vivax* infections by age groups (light microscopy and qPCR); (D) Prevalence of mixed infections by age groups (light microscopy and qPCR). Prevalence data are shown in percentage and 95% confidence intervals.

Furthermore, from the 2,908 samples with DNA material for qPCR analysis, species differentiation showed 162 (37.3%) cases of *P*. *vivax* mono-infection, 151 (34.8%) of *P*. *falciparum* mono-infection, 13 (3.0%) of *P*. *malariae* mono-infection, 1 (0.2%) of *P*. *ovale* mono-infection, and 107 (24.6%) of mixed infection. Mixed infections included mostly the main two species, with 83 cases positive for *P*. *falciparum* and *P*. *vivax*, 15 for *P*. *falciparum* and *P*. *malariae*, 2 for *P*. *falciparum* and *P*. *ovale*, 1 for *P*. *vivax* and *P*. *malariae*, 1 for *P*. *vivax* and *P*. *ovale*, and 1 for *P*. *malariae* and *P*. *ovale*. In addition, there were 3 cases of a triple combination of species, which included 2 cases of *P*. *falciparum*, *P*. *vivax* and *P*. *malariae*, and 1 case of *P*. *vivax*, *P*. *malariae and P*. *ovale*. There was 1 infected case that included the four species. There were three positive samples in the generic quantitative PCR (QMAL) that were negative for the species-specific quantitative PCRs. Positive cases for *P*. *falciparum*, *P*. *vivax* or mixed infections detected by qPCR compared with microscopy and age distribution are shown in Fig 1.

### Factors associated with malaria infection

Associations of the population characteristics with prevalence of *Plasmodium* infection by qPCR are shown in Table 1. Prevalence was higher in males compared to females [17.6% (95% CI: 15.5%-19.6%) vs 12.9% (95% CI: 11.2%-14.5%), p = 0.0004; Chi-squared test], and also varied across age groups, with higher prevalence in children younger than 15 years-old (p<0.0001). On the other hand, malaria prevalence was higher in those participants with lower haemoglobin levels (p = 0.0197). Malaria prevalence by qPCR was also higher in those children with splenomegaly compared to those without [38.5% (95% CI: 23.2%-53.7%) vs 14.3% (95% CI: 11.1%-17.4%), p<0.001], and in pregnant women compared to the non-pregnant women at childbearing age [19.4% (95% CI: 9.5%-29.2%) vs 11.0% (95% CI: 8.9%-13.1%), p = 0.048]. On the other hand, presenting a body temperature of ≥37.8°C was not associated with a higher malaria prevalence (p = 0.5623; Fisher exact test).

**Table 1:** Variables associations with *Plasmodium* infection by qPCR and logistic regression models for risk factors.

Variable		qPCR positive N (%; 95% CI)	p-value	Univariable analysis OR (95% CI)	p-value	Multivariable analysis aOR (95% CI)^#^	p-value
Sex (N = 2908)^a^	Male	233 (17.6; 15.5–19.6)		reference group	0.0004	reference group	0.0103
	Female	204 (12.9; 11.2–14.5)		0.69 (0.56–0.85)		0.75 (0.60–0.93)	
Age (years) (N = 2908)^a^	< 5	63 (17.5; 13.6–19.5)		reference group	< 0.0001	reference group	< 0.0001
	≥ 5 to < 15	128 (21.1; 17.9–24.4)		1.26 (0.90–1.76)		1.33 (0.93–1.89)	
	≥ 15 to < 25	102 (16.6; 13.6–19.5)		0.93 (0.66–1.32)		1.00 (0.69–1.44)	
	≥ 25 to < 35	71 (12.0; 9.3–14.6)		0.64 (0.44–0.92)		0.67 (0.46–1.00)	
	≥ 35 to < 45	31 (9.4; 6.2–12.5)		0.49 (0.31–0.77)		0.50 (0.31–0.81)	
	≥ 45	42 (10.4; 7.4–13.4)		0.55 (0.36–0.83)		0.52 (0.34–0.81)	
Origin (N = 2905)^b^	Born outside Lihir	123 (11.6; 9.7–13.6)		reference group	0.0001	reference group	0.7364
	Born in Lihir	314 (17.0; 15.3–18.7)		1.55 (1.24–1.94)		0.96 (0.73–1.24)	
Geographic area (N = 2908)^a^	Aniolam MIZ	121 (8.5; 7.0–9.9)		reference group	< 0.0001	reference group	< 0.0001
	Aniolam non-MIZ	263 (27.0; 24.2–29.8)		3.99 (3.16–5.04)		3.56 (2.72–4.65)	
	Malie Island	17 (14.4; 8.1–20.7)		1.82 (1.05–3.14)		1.83 (1.04–3.21)	
	Masahet Island	21 (8.6; 5.1–12.2)		1.02 (0.63–1.66)		1.13 (0.68–1.89)	
	Mahur Island	15 (10.4; 5.4–15.4)		1.26 (0.71–2.21)		1.36 (0.75–2.49)	
Type of house (N = 2900)^c^	Permanent	226 (14.4; 12.7–16.1)		reference group	< 0.0001	reference group	0.0017
	Traditional	84 (25.4; 20.7–30.1)		2.04 (1.54–2.71)		1.65 (1.21–2.25)	
	Makeshift	127 (12.7; 10.6–14.8)		0.87 (0.69–1.10)		0.93 (0.72–1.19)	
Living with a malaria positive person (N = 2908)^a^	No	196 (10.3; 9.0–11.7)		reference group	< 0.0001	reference group	< 0.0001
	Yes	241 (23.8; 21.1–26.4)		2.70 (2.20–3.32)		1.94 (1.56–2.42)	
Slept indoors the previous night (N = 2905)^b^	No	24 (17.8; 11.3–24.2)		reference group	0.3627	reference group	0.4349
	Yes	413 (14.9; 13.6–16.2)		0.81 (0.51–1.28)		0.82 (0.50–1.35)	
Slept under LLIN the previous night (N = 2905)^b^	No	268 (14.7; 13.0–16.3)		reference group	0.4528	reference group	0.3085
	Yes	169 (15.7; 13.5–17.9)		1.08 (0.88–1.34)		0.88 (0.70–1.12)	
Haemoglobin (g/dL) (N = 1877)^d^	< 8.0	28 (22.0; 14.8–29.3)	0.0197				
	≥ 8.0 to < 10.0	69 (16.0; 12.5–19.5)					
	≥ 10.0 to < 12.0	107 (14.0; 11.5–16.5)					
	≥ 12.0	66 (11.9; 9.2–114.6)					
Splenomegaly (N = 497)^e^	Yes (Hackett 1–5)	15 (38.5; 23.2–53.7)	<0.001				
	No	68 (14.3; 11.1–17.4)					

Abbreviations: aOR = adjusted odds ratio, LLIN = Long-lasting insecticidal-treated net, MIZ = mine-impacted zone, OR = odds ratio, PNG = Papua New Guinea, qPCR = quantitative polymerase chain reaction. Missing data: ^a^0% missing, ^b^0.1% missing, ^c^0.3% missing, ^d^35.5% missing, ^e^48.6% missing in the studied subgroup (population <15 years-old). ^#^Multivariable analysis conducted for all variables in the table except haemoglobin and splenomegaly, including 2,906 observations.

**Fig 2:**
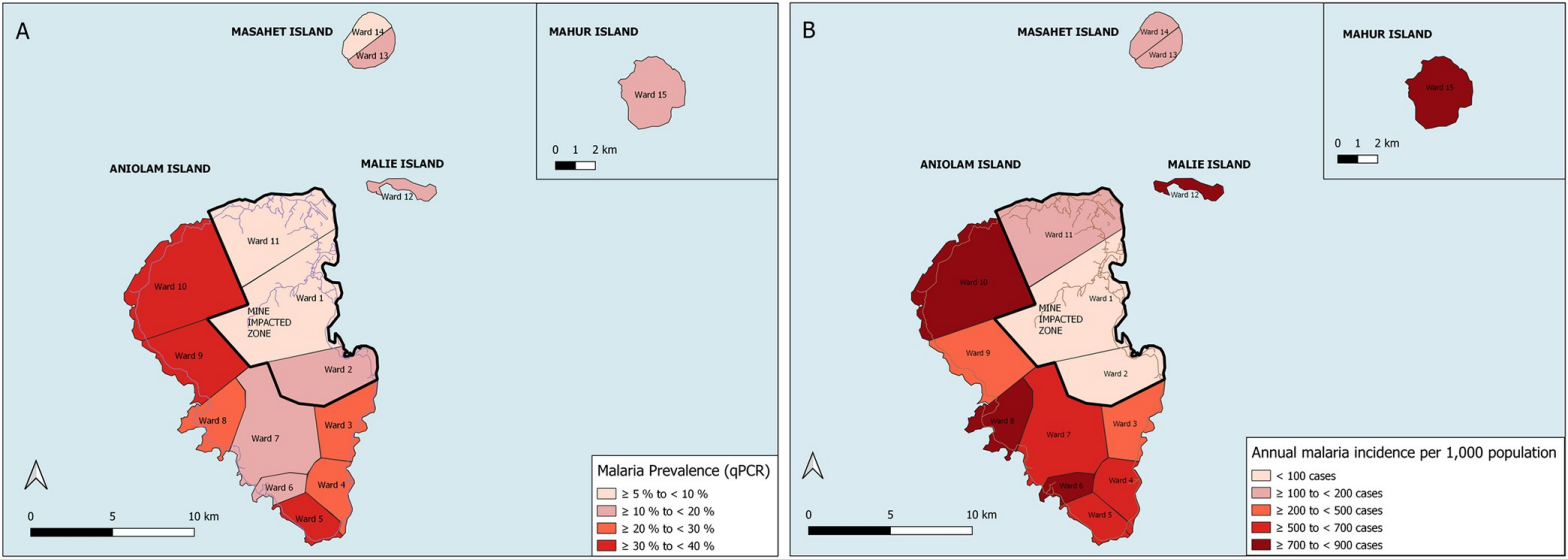
Malaria prevalence and incidence in each geographic area of the Lihir Islands. (A) Malaria prevalence by qPCR presented in percentages, with the denominator being the number of participants included in each area; (B) Incidence rates presented in annual cases per 1,000 inhabitants, with the denominator being the total population of each area (population census conducted in 2018–2020). In both maps, areas are divided by wards (administrative divisions), and the mine impacted zone comprises wards 1, 2 and 11. These maps were created with the software QGIS version 3.16 Hannover. For the base layer we used the country and region limits from OpenStreetMap; map data copyrighted OpenStreetMap contributors and available from https://www.openstreetmap.org. The limits of the mine impact zone and the limits of the administrative wards were plotted through GPS points obtained by the authors of this manuscript. The roads’ shapefile was obtained from the Papua New Guinea Environment Data Portal, an open source available at https://png-data.sprep.org/dataset/png-roads.

Regarding the geographic distribution, prevalence of both microscopic and sub-microscopic infections varied across areas (p<0.0001). The lowest prevalence (qPCR) was found in the MIZ of Aniolam (8.5%; 95% CI: 7.0%-9.9%) and in Masahet Island (8.6%; 95% CI: 5.1%-12.2%), followed by Mahur Island (10.4%; 95% CI: 5.4%-15.4%), Malie Island (14.4%; 95% CI: 8.1%-20.7%) and the non-MIZ of Aniolam (27.0%; 95% CI: 24.2%-29.8%). Of note, we found highly heterogeneous malaria prevalence across villages (S2 Table) and administrative divisions, especially in the non-MIZ, ranging from 12.4% in the south-western coast to 38.9% in the north-western coast of Aniolam. Distribution of positive cases by qPCR across all administrative divisions is shown in Fig 2A.

We performed uni- and multivariable logistic regression analyses to identify the risk associated with being positive for *Plasmodium* parasites by qPCR (Table 1). Haemoglobin levels, splenomegaly, and presence of fever were excluded of the analysis. In the multivariable analysis, the strongest independent risk factor for carrying *Plasmodium* parasites was the geographic area, with the highest risk in the non-MIZ of Aniolam, presenting an adjusted OR (aOR) of 3.56 (95% CI: 2.72–4.65). Sex was also independently associated with parasite prevalence, with an aOR of 0.75 (95% CI: 0.60–0.93) in females. Risk of malaria infection also decreased with age older than 35 years-old (p<0.0001). Finally, other independent risk factors associated with parasite prevalence by qPCR were living with a malaria positive individual [aOR = 1.94 (95% CI: 1.56–2.42; p < 0.0001)] and living in a traditional type of house [aOR = 1.65 (95% CI: 1.21–2.25; p = 0.0017)].

### Passive case detection and malaria incidence in the Lihir Islands

In 2019, a total of 18,419 patients were attended in the Lihirian health facilities with suspected malaria, of whom 9,207 (50%) were considered positive. Of the positive, 7,774 (84.4%) were diagnosed by RDT, 1,377 (15.0%) by microscopy, and 56 (0.6%) based solely on clinical grounds, without using diagnostic tools. Considering only the confirmed cases with microscopy or RDT (total of 9,151), malaria incidence in Lihir was 345 cases per 1,000 inhabitants, with an annual blood examination rate of 69.2%.

Incidence was similar across months (p = 0.054; Mann-Kendall test), with a mean (±SD) of 767.3 (±169.7) cases per month (S2C Fig). Similar to parasite prevalence, incidence varied substantially by geographic areas (p<0.001; Wald test, Fig 2B). In addition, incidence varied across age (p<0.001), with 53.6% of malaria cases occurring in children below 15 years-old (S2A Fig). Incidence rates and incidence risk ratios between the different geographic areas and age groups is shown in S3 Table. Incidence of each *Plasmodium spp*. varied depending on the diagnostic tool used. A comparison of diagnosed *Plasmodium* species by microscopy and RDT is shown in S2B Fig.

### Larval habitats surveillance

**Fig 3:**
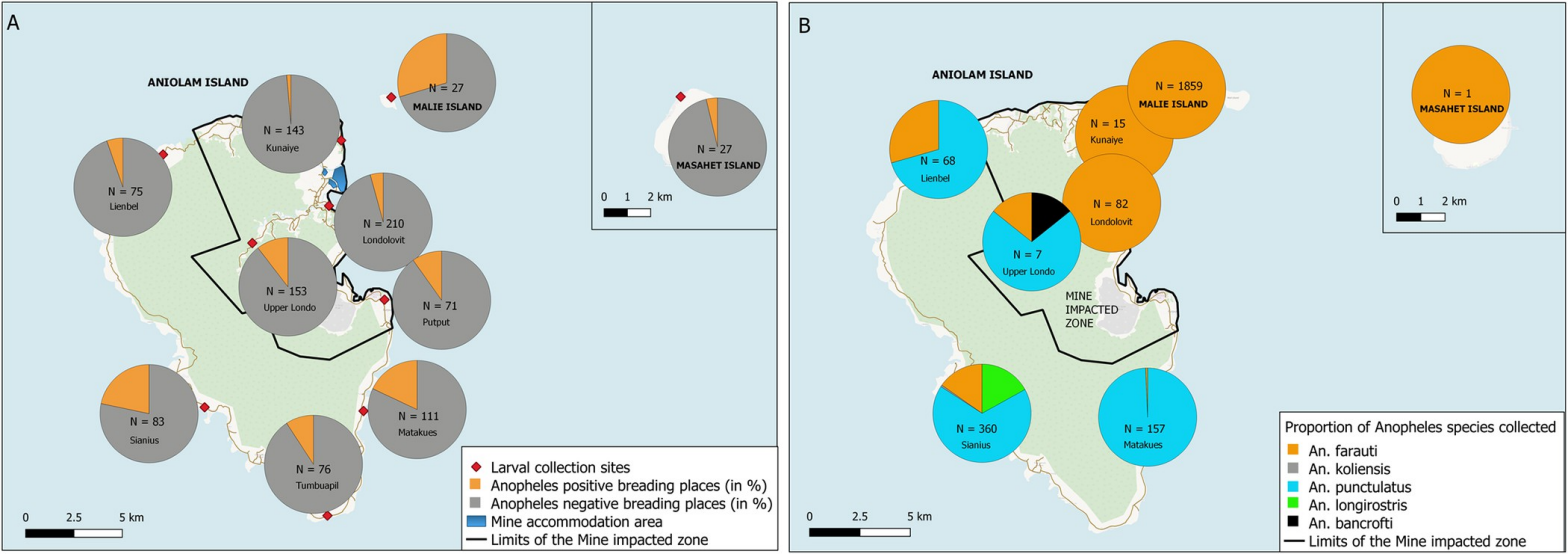
*Anopheles* species and larval habitats in the Lihir Islands. (A) proportion of *Anopheles* positive larval habitats after entomological identification of larvae grown into adults in the insectary, the number of total potential habitats surveyed and the name of each collection site is described in each chart; (B) proportions of each species of *Anopheles* collected in human landing catches measured by qPCR, the number of total *Anopheles* collected per site and the name of each collection site is described in each chart. These maps were created with the software QGIS version 3.16 Hannover. For the base layer we used the country and region limits from OpenStreetMap; map data copyrighted OpenStreetMap contributors and available from https://www.openstreetmap.org. The limits of the mine impact zone and the limits of the administrative wards were plotted through GPS points obtained by the authors of this manuscript. The roads’ shapefile was obtained from the Papua New Guinea Environment Data Portal, an open source available at https://png-data.sprep.org/dataset/png-roads.

A total of 976 potential larval habitats were surveyed at ten entomological surveillance sites (i.e., around the same eight villages used for adult mosquito population, plus two extra villages). Overall, 92 (9.4%) of surveyed habitats were positive for *Anopheles* larvae. The distribution of the larval habitats surveyed and those positive for *Anopheles* larvae is shown in Fig 3A. The proportion of habitats positive for *Anopheles* larvae varied across geographic areas (p<0.0001; Chi-squared test), with Malie Island having the highest (29.5%), followed by the non-MIZ of Aniolam (14.2%), the MIZ (5.9%) and Masahet Island (3.9%), corresponding well with the observed abundance of adult *Anopheles* mosquitoes. The most frequent habitat for *Anopheles* larvae was the permanent groundwater (e.g., swamps), with 25% of the sampled wells positive for *Anopheles* species, followed by transient puddles (16.7%), and forest swamps (15.4%). *Anopheles* species present in each surveyed habitat are shown in S4 Table. It should be noted that the number of Anopheles -positive habitats was small and as such, we were not able to determine if the above proportions were statistically significantly different.

### Adult vector indicators

A total of 2,549 *Anopheles* mosquitoes were collected with HLCs at the eight villages and over 7,821 person-hours of collection. Of these, 2,034 (79.8%) were *An*. *farauti*, 448 (17.5%) were *An*. *punctulatus*, 61 (2.4%) were *An*. *longirostris*, 2 (0.08%) were *An*. *koliensis*, and 1 (0.04%) was *An*. *bancroftii*. There were 3 (0.1%) *Anopheles* mosquitoes collected for which the species could not be ascertained morphologically.

Vector species across the eight HLC collection villages are shown in Fig 3B. Only 1 anopheline specimen was collected in Masahet (*An*. *farauti)*. *S*pecies distribution differed across geographic areas (p<0.0001), with *An*. *farauti* being the predominant vector in the MIZ of Aniolam (94.2%, 95% CI: 88.0–97.3), in Malie Island (100%, 95% CI: 89.9–100.0), and in Masahet Island (n = 1). In contrast, *An*. *punctulatus* was the predominant species in the non-MIZ of Aniolam (75.7%, 95% CI: 72.1–79.0), followed by *An*. *farauti* (13.0%, 95% CI: 10.5–16.0) and *An*. *longirostris* (10.4%, 95% CI: 8.2–13.2).

Human biting rates, sporozoite rates, and the resulting entomological inoculation rates are shown in detail in Table 2. The HBR amongst all *Anopheles* collected (any species) varied significantly across areas (p<0.0001; Chi-squared test), with a mean of 8,487.5 bites per person-year in Malie Island, 896.4 bites per person-year in the non-MIZ, and 175.9 bites per person-year in the MIZ of Aniolam.

From the total of 2,549 *Anopheles* mosquitoes, we tested 1,511 for *Plasmodium* species by qPCR. It was not possible to extract DNA from 5 (6%) mosquitoes collected in the MIZ of Aniolam and 96 (16%) mosquitoes from the non-MIZ area. Since the number of Anopheles mosquitoes was extremely high in Malie (1859) compared to the rest of the Islands and all were *An*. *farauti*, we selected the 50% (924 mosquitoes) from each collection day for undertaken the PCR analysis. From the 1,511 *Anopheles* tested, 1,083 were *An*. *farauti* (53% of all *An*. *farauti* collected), 368 were *An*. *punctulatus* (82% of all *An*. *punctulatus* collected), 57 were *An*. *longirostris* (93% of all *An*. *longirostris* collected), and 2 were *An*. *koliensis* (100% of all *An*. *koliensis* collected). Of these, a total of 108 (7.1%) mosquitoes were positive for *Plasmodium spp* sporozoites. Overall, the sporozoite rate was 4.1% (95% CI: 3.1–5.1) for *P*. *vivax*, 2.5% (95% CI: 1.7–3.3) for *P*. *falciparum*, and 0.5% (95% CI: 0.0–0.9) for mixed infections containing both *Plasmodium* species. The vastly different biting rates within the three geographical regions surveyed resulted in very different EIR estimates, with Malie Island exhibited the highest EIR estimates, followed by the non MIZ of Aniolam, and the MIZ having the lowest EIR estimates (Table 2).

**Table 2:** Summary of adult vector indicators in each geographic area.

Collection site	Vector	HBR (95% CI)	All *Plasmodium* species		*Plasmodium falciparum*		*Plasmodium vivax*		Mixed infections	
			SR (95% CI)	EIR (95% CI)	SR (95% CI)	EIR (95% CI)	SR (95% CI)	EIR (95% CI)	SR (95% CI)	EIR (95% CI)
Aniolam MIZ	*An*. *farauti*	165.7 (140.5–190.9)	16.3% (8.8–23.9)	27.0 (12.4–45.4)	3.3% (0.0–6.9)	5.5 (0.0–13.2)	12.0% (5.3–18.6)	19.9 (7.4–35.5)	1.1% (0.0–3.2)	1.8 (0.0–6.1)
	*An*. *punctulatus*	8.5 (2.8–14.2)	0.0% (0.0–0.0)	0 (0.0–0.0)	0.0% (0.0–0.0)	0 (0.0–0.0)	0.0% (0.0–0.0)	0 (0.0–0.0)	0.0% (0.0–0.0)	0 (0.0–0.0)
	All *Anopheles*	175.9 (149.9–201.9)	15.3% (8.2–22.4)	26.9 (12.3–45.2)	3.1% (0.0–6.5)	5.5 (0.0–13.1)	11.2% (5.0–17.5)	19.7 (7.9–35.3)	1.0% (0.0–3.0)	1.8 (0.0–6.1)
Aniolam non-MIZ	*An*. *farauti*	117.2 (96.0–138.4)	13.4% (5.3–21.6)	15.7 (5.1–29.9)	3.0% (0.0–7.1)	3.5 (0.0–9.8)	10.4% (3.1–17.8)	12.3 (3.0–24.6)	0.0% (0.0–0.0)	0 (0.0–0.0)
	*An*. *punctulatus*	681.8 (630.6–733.0)	8.3% (5.4–11.1)	56.6 (34.1–81.4)	1.4% (0.2–2.6)	9.5 (1.3–19.1)	6.3% (3.8–8.8)	43.0 (24.0–65.2)	0.6% (0.0–1.3)	4.1 (0.0–9.5)
	*An*. *longirostris*	92.8 (73.9–111.7)	10.5% (2.6–18.5)	9.7 (1.9–20.7)	0.0% (0.0–0.0)	0 (0.0–0.0)	10.5% (2.6–18.5)	9.7 (1.9–20.7)	0.0% (0.0–0.0)	0 (0.0–0.0)
	All *Anopheles*	896.4 (837.7–955.1)	9.2% (6.6–11.8)	82.5 (55.3–112.7)	1.4% (0.4–2.5)	12.5 (3.4–23.9)	7.4% (5.0–9.7)	66.3 (41.9–92.6)	0.4% (0.0–1.0)	3.6 (0.0–9.6)
Malie Island	*An*. *farauti*	8487.5 (8306.9–8668.1)	5.2% (3.8–6.6)	441.3 (315.7–572.1)	3.0% (1.9–4.1)	254.6 (157.8–355.4)	1.6% (0.8–2.4)	135.8 (66.5–208.0)	0.5% (0.1–1.0)	42.4 (8.3–86.7)
	All *Anopheles*	8487.5 (8306.9–8668.1)	5.2% (3.8–6.6)	441.3 (315.7–572.1)	3.0% (1.9–4.1)	254.6 (157.8–355.4)	1.6% (0.8–2.4)	135.8 (66.5–208.0)	0.5% (0.1–1.0)	42.4 (8.3–86.7)

Legend: Human biting rate (expressed in bites per person-year), sporozoite rate (percentage of sporozoite infected mosquitoes) and entomological inoculation rate (percentage of infective bites per person-year) are shown in proportions and 95% confidence intervals (CI) per each of the species of *Anopheles* collected, *Plasmodium* species, and geographical areas in Lihir Islands. Masahet was excluded from this analysis as only 1 *Anopheles* was collected. Abbreviations: EIR = entomological inoculation rate, HBR = Human Biting Rate, MIZ = mine-impacted zone, SR = sporozoite rate.

Only 26.3% of mosquitoes were captured indoors despite balanced collection effort indoors and outdoors, with *An*. *farauti* (26.5%), *An*. *punctulatus* s.s. (25%), and *An*. *longirostris* (26.2%) all exhibiting similar preference for outdoor biting. There was no apparent difference in the preference for outdoor biting in the different geographical zones.

**Fig 4:**
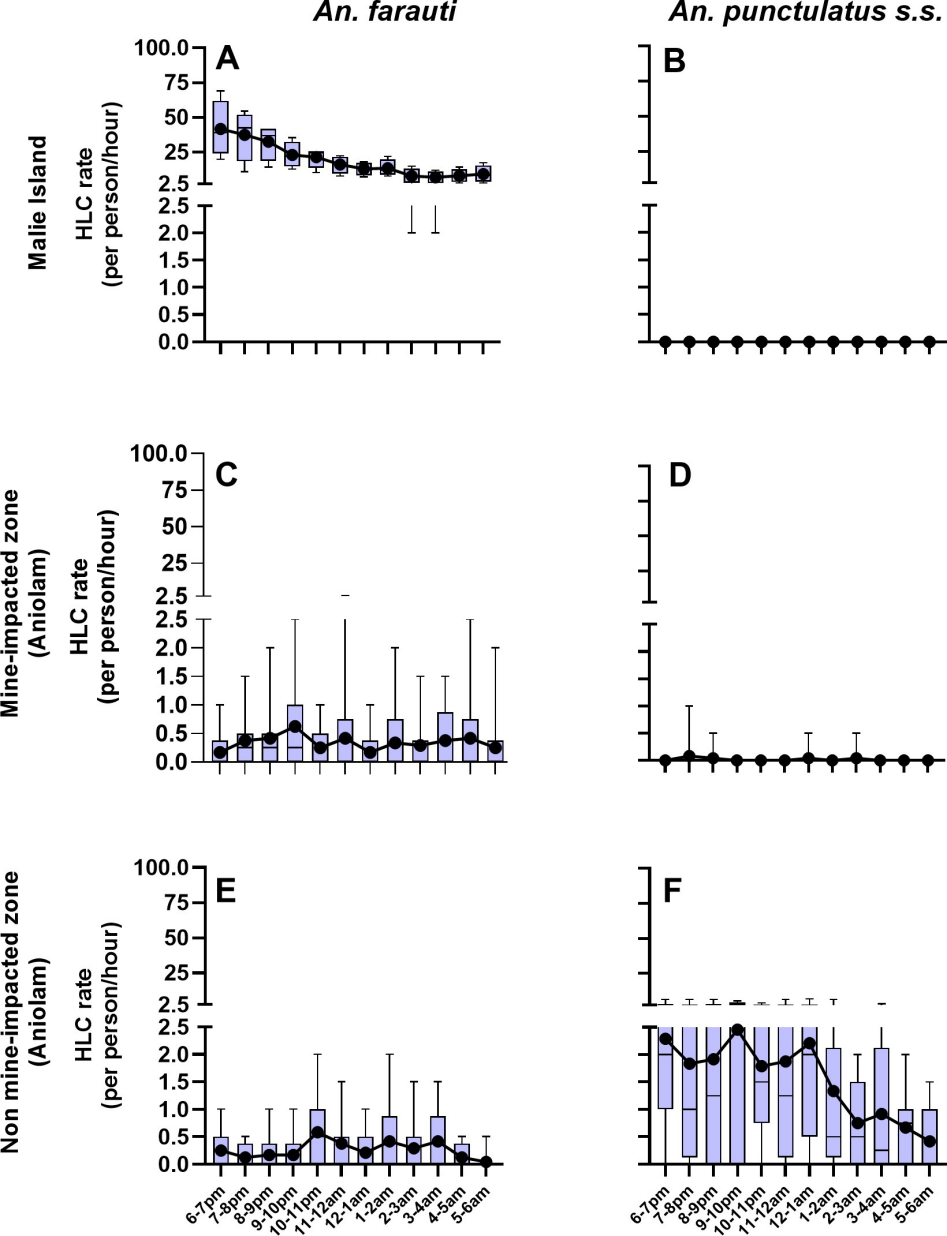
Human landing catch rates for the major vectors found in the Lihir Islands. The graphs use combined indoor and outdoor HLC rates for each night of collection per zone (Malie Island: 4 nights; MIZ and non-MIZ of Aniolam: 12 nights). (A) and (B), Malie Island; (C) and (D), the Mine impacted zone (MIZ) of Aniolam; (E) and (F), Aniolam out of the Mine impacted zone (Aniolam non-MIZ). The box and whisker plots present the median, interquartile ranges and ranges of the catch rates. The black dots represent the mean of the catch rates. Both statistics are presented to account for the highly skewed nature of the data.

Biting behaviour over the course of the night was only analysed for the two most abundant *Anopheles* species, *An*. *farauti and An*. *punctulatus* s.s, as shown in Fig 4. *An*. *farauti* on Malie Island exhibited an early biting behaviour with a peak at 6-7pm. *An*. *punctulatus*, most abundant in the non-MIZ of Aniolam, exhibited the typical late-biting profile characteristic for this species. The very low abundance of either *Anophele*s species in the other zones precluded an accurate determination of their biting profiles in these zones.

### Correlation between vector and human indicators

We assessed the relationship between HBR and EIR with malaria prevalence in each of the villages where both entomological and human indicators were collected; as well as the relationship between EIR and incidence data from the month after the collection. Data on human and vector indicators at village level are shown in Table 3. Although not statistically significant, there was a clear trend towards a positive correlation between the HBR and the incidence (rho = 0.63, 95% CI: -0.15,0.93; Spearman test), as well as between the EIR and the incidence (rho = 0.52, 95% CI: -0.32,0.90) with the village in Malie being the one with higher entomological indicators and higher malaria incidence, and Upper Londolovit in the MIZ on the opposite position. On the other hand, there was no association between the entomological indicators and the prevalence of *Plasmodium spp* by qPCR. The results of this analysis can be seen in S3 Fig.

**Table 3:** Summary of vector and human metrics in each of the entomological sites.

Geographic area	Village	Vector metrics			Human metrics	
		SR	HBR	EIR	Prevalence	Incidence
Aniolam MIZ	Londolovit	0.207	534.83	110.88	8.4%	1.1
	Upper Londo	0	31.96	0	8.5%	1.1
	Kunaye 2	0	70.24	0	18.1%	14.1
Aniolam non-MIZ	Lienbel	0.053	318.42	16.76	38.5%	42.9
	Matakues	0.077	716.8	55.14	32.8%	20.3
	Sianius	0.115	1664.43	190.92	20.0%	18.9
Malie Island	Malie (Sinambiet)	0.063	8487.5	532.76	14.4%	87.5
Masahet Island	Masahet (Ton)	0	4.57	0	9.5%	9.7

Legend: Vector metrics such as human biting rate (in bites per person-year), sporozoite rate and entomological inoculation rate (percentage of infective bites per person-year), and human prevalence (percentage of infected individuals in the cross-sectional survey) and incidence of the month of December 2019 (number of cases per 1,000 inhabitants) for each of the villages with entomological surveillance. Abbreviations: EIR = entomological inoculation rate, HBR = Human Biting Rate, MIZ = mine-impacted zone, SR = sporozoite rate.

## Discussion

This study characterized the human and vector determinants behind the different intensities of malaria transmission on the Lihir Islands of PNG, which are influenced by a gold mining operation. We showed that the MIZ of Aniolam, exhibiting a prevalence of 5.8% in children by light microscopy in 2010 (18), maintained this reduced burden with similar prevalence rates. However, there has been a three-fold decrease in infection incidence in this area over the past decade (from 437 cases per 1,000 inhabitants in 2011, to 142 cases per 1,000 inhabitants in 2019). In contrast, the non-MIZ of Aniolam and Malie Island exhibited significantly higher infection rates rarely seen in PNG [9,15,23]. Notably, the north-western zone of Aniolam showed prevalence rates similar to those observed in heavily malaria-burdened countries in Africa [3]. In this context, the study population, especially children, presented characteristics associated with high malaria exposure, such as moderate and severe anaemia, and splenomegaly [24]. These clinical characteristics were more common outside the MIZ and were associated with carrying malaria parasites. Indeed, the strongest independent risk factor for carrying malaria parasites was the geographic location of the participants’ households. The decrease in malaria burden in the MIZ of Aniolam over the last decades can be attributed to different reasons. First, an improved socioeconomic profile due to higher employment rate in the area, along with improved housing conditions, including more permanent houses. Second, enhanced access to quality healthcare, since the hospital run by the mine rarely experiences stock outs and is better equipped than other health facilities. Finally, implementation of vector control strategies by the mine company since 2006, including the distribution of mosquito nets, larviciding, and environmental management to remove breeding sites, while other areas of Lihir received only mosquito nets from 2009 onwards. A previous study highlighted substantial differences in prevalence within the MIZ since the beginning of the mine vector control programme (31.5% of the children surveyed in 2005 compared to 5.8% in 2010) [18], which likely reflect the impact of the vector control as well as the other mentioned improvements within the MIZ. The same study reported a minor decrease in prevalence outside the MIZ at the same time points (from 34.9% of prevalence in children to 26.9%).

As this study was conducted at the household level, we were able to demonstrate that, after geographic location, sharing a household with a positive individual was the strongest risk factor for malaria infection. This finding aligns with other studies conducted in Equatorial Guinea and Kenya [25,26]. Additionally, living in a traditional house was associated with a higher risk of infection, attributed to the open housing structures inherent in traditional dwellings, known to exacerbate malaria transmission [27]. Of note, use of LLIN was low across the Lihir Islands, with only 37.2% of the participants sleeping under an LLIN the previous night. Although this percentage is higher than a previous study reporting a mere 13.6% LLIN use [19], LLINs coverage remained insufficient. Also, we did not observe any association between their use and the prevalence of infection, since infection may depend on many other factors such as outdoors transmission, early mosquito biting times, or entomological indicators.

*P*. *falciparum* and *P*. *vivax* were the most prevalent species identified in both the prevalence survey and the passive case detection analyses. Mixed infections were detected in a quarter of the positive cases when using qPCR, mainly due to the revealed *P*. *vivax* submicroscopic infections, and they were more frequent in children. These findings are consistent with reports from other regions of PNG and the Solomon Islands [28,29]. A previous study in PNG demonstrated that one of the factors contributing to this higher rate of *P*. *vivax* sub-microscopic infections in children is the occurrence of frequent relapses [30]. A limitation of our study stems from assessing prevalence only once, hence not considering climatic patterns throughout the year. However, incidence of malaria in Lihir during 2019 showed no significant variations across months. Of note, we observed few discrepancies between microscopy and RDT in the prevalence survey. We could explain the additional *P*. *falciparum* infections detected by RDT due to persistence of HRP2 antigen during antimalarial treatment, while the non-*falciparum* infections missed by RDT were attributed to the lower sensitivity of this tool in detecting these species compared to microscopy [31]. On the other hand, the incidence data showed a higher number of mixed infections when using RDT, probably due to some pLDH lines caused by high *P*. *falciparum* parasitaemia [32].

The entomology surveys confirmed a worryingly high outdoor and early biting feeding behaviour for the anopheline populations, particularly *An*. *faurauti*, consistent with previous findings in PNG [15]. While a study conducted in different villages of two provinces with moderate-to-high malaria transmission showed no increase in the proportion of infective bites occurring before 10 pm [17], a subsequent study conducted in Madang Province highlighted the epidemiological significance of this earlier feeding behaviour by *An*. *farauti* [33]. This discovery challenges the effectiveness of universal coverage with LLINs as the main, and often only, vector control strategy in our setting. People in PNG often spend a significant portion of their evenings and early nights engaged in outdoor activities without protection [27]. The early biting behaviour of *An*. *farauti* has also been observed in the Solomon Islands [34], emphasizing the need for innovative vector control strategies targeting these species. Hence, our study supports that LLINs alone are insufficient to reduce malaria transmission in the Pacific. In the Lihir Islands vector densities and infection rates are higher in areas of Lihir relying solely on LLINs and case management (non-MIZ of Aniolam and Malie Island), compared to the areas benefiting from alternative vector control interventions implemented by the mining operator.

Moreover, vector composition, proportion of larvae colonized sites, and transmission intensities were also different across the areas, aligning with malaria burden variations as seen in the correlation analysis, especially for the incidence data. Despite the open-pit mine and the abundant human dwellings in the MIZ of Aniolam, larvae colonized sites and HBR/EIR are low, possibly due to the specific larviciding conducted in this area [35], as well as better housing conditions [36]. Conversely, Malie Island exhibits the highest HBR and EIR among the Lihir Islands, with most of the breeding sites found colonized by anophelines. The numerous mangroves located in this area may contribute to the highest density of *An*. *farauti*, as it is known to breed on brackish water and coastal streams [37]. In contrast, the non-MIZ of Aniolam exhibits a diverse mosquito ecology with *An*. *punctulatus* as the main vector, which is considered more efficient [38] and potentially more anthropophilic [39]. The environmental changes on Aniolam, including road extension, may explain the predominant presence of *An*. *punctulatus* outside the MIZ [40]. Surprisingly, Masahet Island, relying solely on LLINs for vector control, had the lowest mosquito and larvae densities, along with a lower human burden. Masahet Island has reduced mangroves and swamps areas, and villages are coastal, with residents keeping livestock fenced several metres away from houses, unlike other areas of the Lihir Islands. A link between proximity of cattle to human dwellings and a higher risk of infection has been showed before [41]. Hence, this distinct behaviour could explain the lower vector densities on Masahet Island, especially as the only vector identified there was *An*. *farauti*, a highly generalist mosquito [39].

Identifying human and vector factors for malaria risk serves for locating areas of concentrated malaria transmission and directing targeted measures to reduce the disease burden [42]. Data on prevalence and incidence of *P*. *vivax* infections, particularly among children, underscore the need to enhance radical cure implementation to prevent relapses and maximize transmission reduction, as recommended by a cohort study in East Sepik [43]. Focusing on the paediatric population is crucial in moderately to highly transmissive settings like Lihir, where interventions targeting transmission reduction would have the most significant impact [44]. It may also be beneficial to explore specific measures for reducing intrahousehold transmission, such as reactive focal mass drug administration at the household and neighbouring levels; even though its effectiveness has been better demonstrated in low-endemicity settings [45]. Despite reported changes in *Anopheles* biting behaviours, it remains advisable to improve the usage of LLINs; however, the decreased bioefficacy of distributed nets in recent campaigns in PNG [46] encourages the need for regular monitoring of this strategy [47] and increase of funding, commitment, and innovative strategies [48]. The evidence of lower malaria burden and reduced entomological metrics in Masahet and the MIZ of Aniolam supports the implementation of specific vector control strategies across the Lihir Islands. This could include measures like segregating livestock from the human population [49], exploring endectocidal treatments such as ivermectin for livestock [50], and expanding larviciding and environmental management to target breeding sites beyond the MIZ [51].

## Conclusion

The current study unveils unique transmission patterns within the Lihir Islands, emphasizing the necessity for customized strategies tailored to the specific characteristics of each area. However, considering the high permeability of these areas, both inside and outside the MIZ, it is crucial for the mine company to strengthen existing malaria control strategies and introduce innovative approaches across the whole group of Islands. This comprehensive analysis holds the potential to guide ongoing malaria control efforts and provide a roadmap for addressing similar challenges in New Ireland Province and other high-transmission coastal zones across the Western Pacific region.

## Supporting information

S1 Fig Map of the Lihir Islands of Papua New Guinea. Located in the New Ireland Province, the Lihir Islands group is formed by three raised coral platform islands and two low coralline islets. The map shows the limits of the mine-impacted zone and the location of all the health facilities inside and outside this area. This map was created with the software QGIS version 3.16 Hannover. For the base layer we used the country and region limits from OpenStreetMap; map data copyrighted OpenStreetMap contributors and available from https://www.openstreetmap.org. The limits of the mine impact zone, the limits of the administrative wards, and the health facilities location were plotted through GPS points obtained by the authors of this manuscript. The roads’ shapefile was obtained from the Papua New Guinea Environment Data Portal, an open source available at https://png-data.sprep.org/dataset/png-roads.
(TIF)

S2 Fig Malaria cases diagnosed at the Lihirian health facilities. (A) Total number of malaria cases by age groups (unspecified group are adults’ patients without identified age); (B): *Plasmodium* species diagnosed by light microscopy and Rapid Diagnostic Test (RDT) in patients presented at the health facilities. For the RDT results, the health facilities recorded *P*. *falciparum* if the test showed a single line for histidine-rich protein 2 (HRP2), non *P*. *falciparum* (*P*. *vivax* in the figure) if the test showed a single line for *Plasmodium* lactate dehydrogenase (pLDH), and mixed infection if the test showed the two lines; (C): Seasonality of malaria infections (Incidence per 1,000 inhabitants) during 2019, with difference between incidences across months of p = 0.054 (Mann-Kendall test).
(TIF)

S3 Fig Correlation analysis between vector and human indicators in the Lihirian villages. (A) Correlation analysis between EIR and prevalence; (B) Correlation analysis between EIR and incidence; (C) Correlation analysis between HBR and prevalence; (D) Correlation analysis between HBR and incidence. HBR are expressed in bites per person-year, EIR are expressed in percentages, prevalence is expressed in percentage, and incidence is expressed in number of cases per 1,000 inhabitants. Abbreviations: CI = confidence interval, EIR = entomological inoculation rate, HBR = Human Biting Rate, MIZ = mine-impacted zone, p = correlation p-value, S = Spearman test value, rho = correlation coefficient.
(TIF)

S1 Table Characteristics of the participants included in the prevalence survey. Abbreviations: MIZ = mine-impacted zone, SD = Standard deviation. ^a^n = 2911 (0.1% missing), ^b^n = 1116 (0% missing, only for females with ≥ 16 years-old), ^c^n = 805 (0% missing, only for participants between 5–18 years-old), ^d^n = 2,912 (0.1% missing), ^e^n = 2,884 (1.0% missing), ^f^n = 1486 (49.0% missing), ^e^n = 1877 (35.6% missing).
(DOCX)

S2 Table Malaria prevalence (by qPCR) found in each of the Lihirian villages. Abbreviations: CI (Confidence Interval), MIZ (Mine-impacted zone). Description of number of inhabitants per village (population census 2018), number of participants per village, and prevalence expressed in % and 95% CI.
(DOCX)

S3 Table Malaria incidence risks in the Lihir Islands by age groups and geographic areas. Abbreviations: CI (Confidence Interval), IR (Incidence Risk), IRR (Incidence Risk Ratio), MIZ (Mine-impacted zone). Binomial regression model was used to estimate IRR with 95% CI and p-values.
(DOCX)

S4 Table Type of habitats where anophelines were found in the Lihir Islands. Abbreviations: CI (Confidence Interval), N (number), Prop (proportion).
(DOCX)

S1 File Supplementary methods. (DOCX)

S1 Data Repository file with the cross-sectional, the incidence, and the entomological data included in this study. (XLSX)
