# Supplementary figures and images for: Human and entomological determinants of malaria transmission in the Lihir Islands of Papua New Guinea: A cross-sectional study
Source: PLoS Negl Trop Dis. 2025 Jan 3;19(1):e0012277. doi: 10.1371/journal.pntd.0012277 (PMC11734946; doi:10.1371/journal.pntd.0012277)

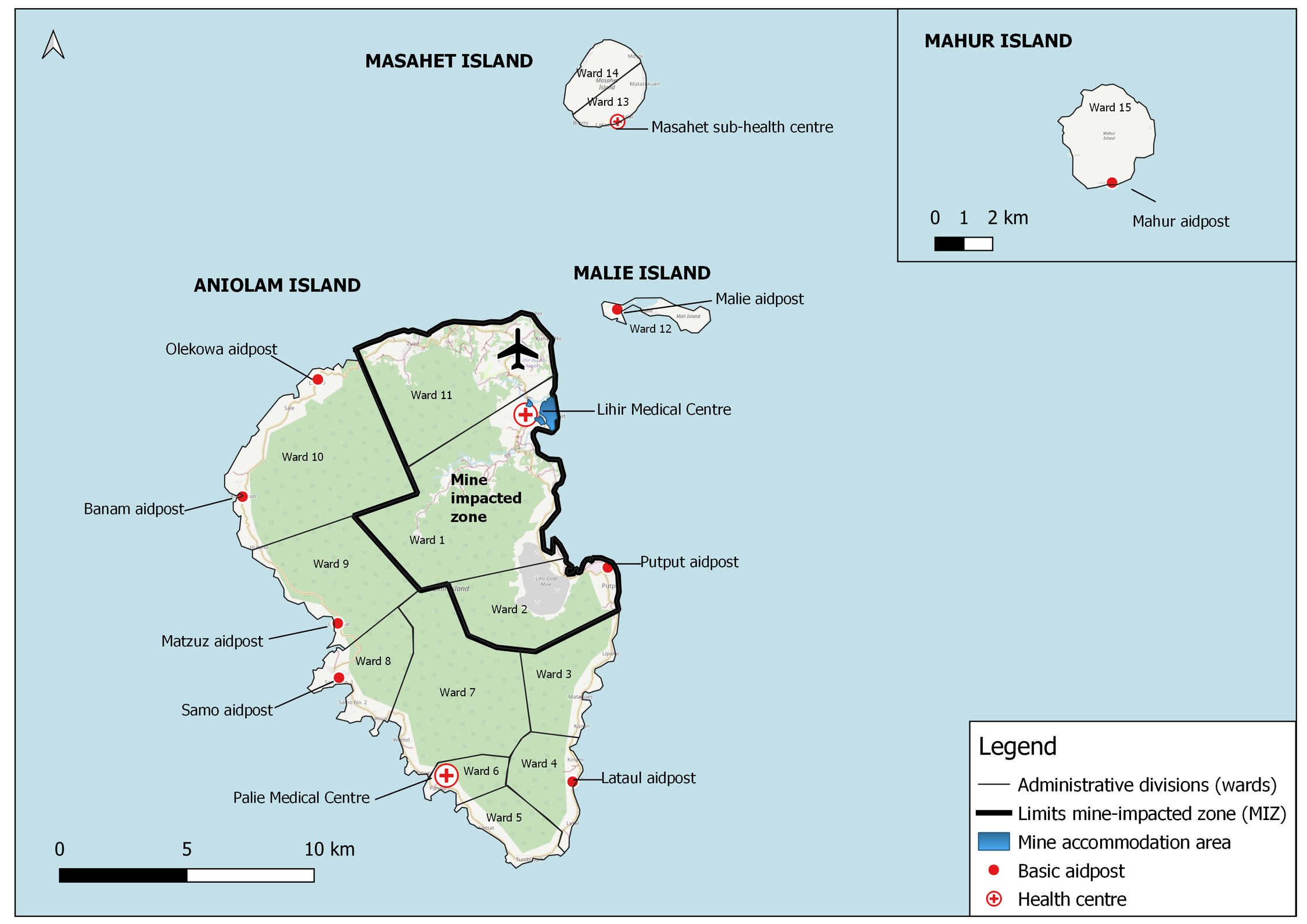

Supplement: S1 Fig — Located in the New Ireland Province, the Lihir Islands group is formed by three raised coral platform islands and two low coralline islets. The map shows the limits of the mine-impacted zone and the location of all the health facilities inside and outside this area. This map was created with the software QGIS version 3.16 Hannover. For the base layer we used the country and region limits from OpenStreetMap; map data copyrighted OpenStreetMap contributors and available from https://www.openstreetmap.org. The limits of the mine impact zone, the limits of the administrative wards, and the health facilities location were plotted through GPS points obtained by the authors of this manuscript. The roads’ shapefile was obtained from the Papua New Guinea Environment Data Portal, an open source available at https://png-data.sprep.org/dataset/png-roads. (TIF) [file pntd.0012277.s001.tif]

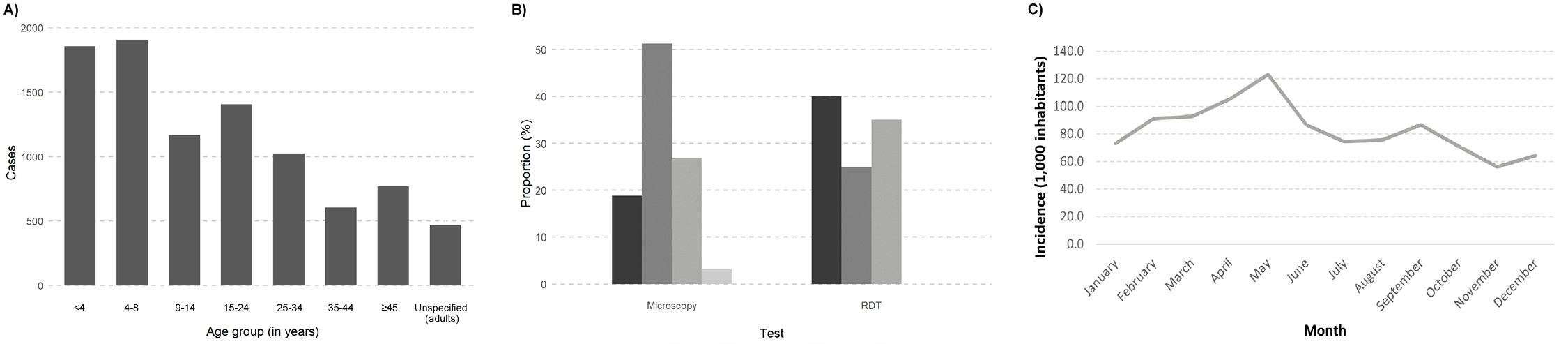

Supplement: S2 Fig — (A) Total number of malaria cases by age groups (unspecified group are adults’ patients without identified age); (B): Plasmodium species diagnosed by light microscopy and Rapid Diagnostic Test (RDT) in patients presented at the health facilities. For the RDT results, the health facilities recorded P. falciparum if the test showed a single line for histidine-rich protein 2 (HRP2), non P. falciparum (P. vivax in the figure) if the test showed a single line for Plasmodium lactate dehydrogenase (pLDH), and mixed infection if the test showed the two lines; (C): Seasonality of malaria infections (Incidence per 1,000 inhabitants) during 2019, with difference between incidences across months of p = 0.054 (Mann-Kendall test). (TIF) [file pntd.0012277.s002.tif]

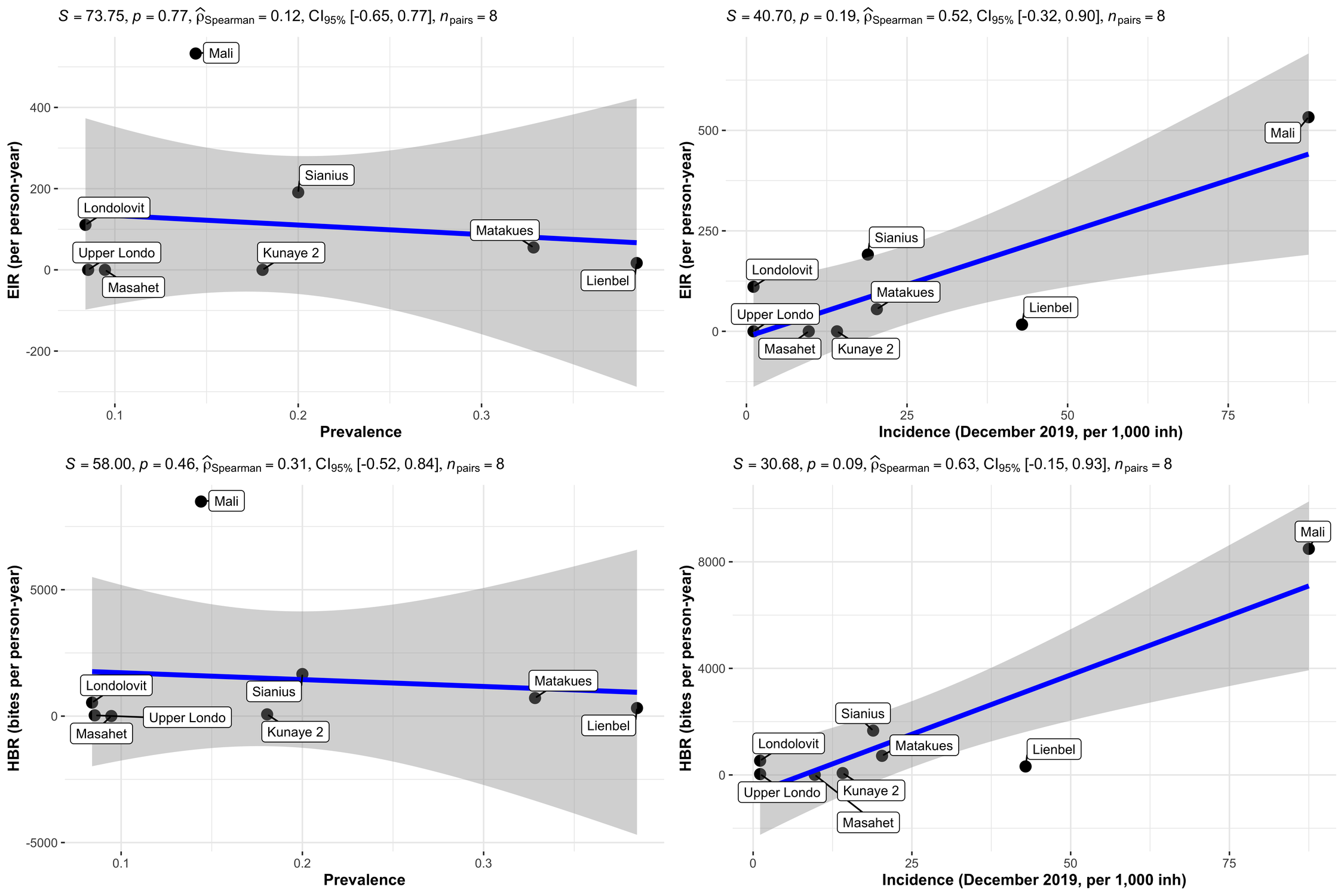

Supplement: S3 Fig — (A) Correlation analysis between EIR and prevalence; (B) Correlation analysis between EIR and incidence; (C) Correlation analysis between HBR and prevalence; (D) Correlation analysis between HBR and incidence. HBR are expressed in bites per person-year, EIR are expressed in percentages, prevalence is expressed in percentage, and incidence is expressed in number of cases per 1,000 inhabitants. Abbreviations: CI = confidence interval, EIR = entomological inoculation rate, HBR = Human Biting Rate, MIZ = mine-impacted zone, p = correlation p-value, S = Spearman test value, rho = correlation coefficient. (TIF) [file pntd.0012277.s003.tif]
