# Supplementary material for: Human and entomological determinants of malaria transmission in the Lihir Islands of Papua New Guinea: A cross-sectional study
Source: PLoS Negl Trop Dis. 2025 Jan 3;19(1):e0012277. doi: 10.1371/journal.pntd.0012277 (PMC11734946; doi:10.1371/journal.pntd.0012277)
Supplement: S1 Table — Abbreviations: MIZ = mine-impacted zone, SD = Standard deviation. an = 2911 (0.1% missing), bn = 1116 (0% missing, only for females with ≥ 16 years-old), cn = 805 (0% missing, only for participants between 5–18 years-old), dn = 2,912 (0.1% missing), en = 2,884 (1.0% missing), fn = 1486 (49.0% missing), en = 1877 (35.6% missing). (DOCX) [file pntd.0012277.s004.docx]

| **Variable** | | **N (%)** |
| --- | --- | --- |
| **Demographic characteristics** | |  |
| Sex (N=2914) | Male | 1327 (45.5) |
|  | Female | 1587 (54.5) |
| Age (years) (N=2914) | < 5 | 361 (12.4) |
|  | 5 to 14 | 606 (20.8) |
|  | 15 to 24 | 615 (21.1) |
|  | 25 to 34 | 595 (20.4) |
|  | 35 to 44 | 332 (11.4) |
|  | ≥ 45 | 405 (13.9) |
| Origin (N=2911) ^a^ | Born in Lihir Islands | 1850 (63.6) |
|  | Born in other PNG regions | 1061 (36.4) |
| Pregnant (N=924) ^b^ | Yes | 62 (6.7) |
| Attending school (N=805) ^c^ | Yes | 492 (61.1) |
| **Geographic characteristics** | | |
| Geographic area in Lihir (N=2914) | Aniolam MIZ | 1433 (49.2) |
|  | Aniolam non-MIZ | 976 (33.5) |
|  | Malie | 118 (4.0) |
|  | Masahet | 243 (8.3) |
|  | Mahur | 144 (4.9) |
| Type of house (N=2912) ^d^ | Permanent | 1580 (54.3) |
|  | Traditional | 333 (11.4) |
|  | Makeshift | 999 (34.3) |
| **Sleeping habits** | | |
| Slept indoors previous night (N=2914) | Yes | 2776 (95.4) |
| Slept under ITN previous night (N=2914) | Yes | 1082 (37.2) |
| **Clinical characteristics** |  |  |
| Presence of fever preceding 2 weeks (N=2911) ^a^ | Yes | 649 (22.3) |
| Temperature (ºC) mean (SD) (N=2884) ^e^ | | 36.4 (0.5) |
| Temperature (ºC) (N=2884) ^e^ | ≥ 37.8 | 23 (0.8) |
| Received antimalarial the previous month (N=2914) | Yes | 325 (11.2) |
| Receiving antimalarial at time of the survey (N=2914) | Yes | 46 (1.6) |
| Splenomegaly (N=516) ^f^ | Yes (Hackett grades 1-5) | 39 (7.6) |
| Haemoglobin (g/dL) (N=1877) ^g^ | < 8.0 | 127 (6.8) |
|  | ≥ 8.0 to < 10.0 | 431 (23.0) |
|  | ≥ 10.0 to < 12.0 | 764 (40.7) |
|  | ≥ 12.0 | 555 (29.6) |

Abbreviations: ITN = insecticide-treated net; MIZ = mine-impacted zone, SD = Standard deviation. Missing data: ^a^0.1% missing, ^b^0% missing in the studied subgroup (only for women between ≥ 16 and < 50 years-old), ^c^0% missing in the studied subgroup (only for participants between 5-18 years-old), ^d^0.1% missing, ^e^1.0% missing, ^f^48.6% missing in the studied subgroup (only for participants being ≤ 15 years-old), ^g^35.6% missing.
