# Supplementary material for: Human and entomological determinants of malaria transmission in the Lihir Islands of Papua New Guinea: A cross-sectional study
Source: PLoS Negl Trop Dis. 2025 Jan 3;19(1):e0012277. doi: 10.1371/journal.pntd.0012277 (PMC11734946; doi:10.1371/journal.pntd.0012277)
Supplement: S2 Table — Abbreviations: CI (Confidence Interval), MIZ (Mine-impacted zone). Description of number of inhabitants per village (population census 2018), number of participants per village, and prevalence expressed in % and 95% CI. (DOCX) [file pntd.0012277.s005.docx]

| **Geographic area** | **Village** | **Number of inhabitants (population census)** | **Number of participants (cross-sectional study)** | **qPCR positive**  **% (95% CI)** |
| --- | --- | --- | --- | --- |
| Aniolam MIZ | Kunaiye 1 | 2284 | 232 | 7.3 (4.0, 10.7) |
|  | Kunaiye 2 | 772 | 79 | 16.5 (8.3, 24.6) |
|  | Khul | 699 | 70 | 4.3 (-0.5, 9.0) |
|  | Zuen | 1830 | 188 | 3.2 (0.7, 5.7) |
|  | Londolovit Town | 413 | 44 | 13.6 (3.5, 23.8) |
|  | Upper Londolovit | 1997 | 204 | 8.3 (4.5, 12.1) |
|  | Lower Londolovit | 1261 | 131 | 8.4 (3.6, 13.1) |
|  | Landolam | 1779 | 183 | 5.5 (2.2, 8.8) |
|  | Potzlaka | 290 | 33 | 6.1 (-2.1, 14.2) |
|  | Marahum | 265 | 32 | 3.1 (-2.9, 9.2) |
|  | Putput 1 | 1253 | 128 | 13.3 (7.4, 19.2) |
|  | Putput 2 | 1026 | 1107 | 16.8 (9.7, 23.9) |
| Aniolam non-MIZ | Lipukuo | 820 | 82 | 9.8 (3.3, 16.2) |
|  | Matakues | 782 | 67 | 32.8 (21.6, 44.1) |
|  | Kanaan | 260 | 31 | 48.4 (30.8, 66.0) |
|  | Kinami | 309 | 39 | 20.5 (7.8, 33.2) |
|  | Lataul | 354 | 30 | 30.0 (13.6, 46.4) |
|  | Lissel | 437 | 63 | 17.5 (8.1, 26.8) |
|  | Tumbuapil | 493 | 49 | 22.4 (10.8, 34.1) |
|  | Komat 1 | 435  399 | 46 | 28.3 (15.2, 41.3) |
|  | Komat 2 |  | 42 | 47.6 (32.5, 62.7) |
|  | Pangoh/Palie | 794 | 82 | 13.4 (6.0, 20.8) |
|  | Talies | 294 | 31 | 19.4 (5.4, 33.3) |
|  | Hurtol | 807 | 58 | 10.3 (2.5, 18.2) |
|  | Sianios | 369 | 40 | 20.0 (7.6, 32.4) |
|  | Samo 1 | 648 | 72 | 31.9 (21.2, 42.7) |
|  | Samo 2 | 320 | 34 | 35.3 (19.2, 51.4) |
|  | Lamboar/Matzuz | 842 | 70 | 28.6 (18.0, 39.2) |
|  | Huniho | 117 | 30 | 60.0 (42.5, 77.5) |
|  | Kosmaiyun | 358 | 39 | 48.7 (33.0, 64.4) |
|  | Sale | 215 | 31 | 25.8 (10.4, 41.2) |
|  | Lienbel | 389 | 39 | 38.5 (23.2, 53.7) |
| Malie Island | Malie | 530  203 | 86 | 12.8 (5.7, 19.8) |
|  | Sinambiet |  | 32 | 18.8 (5.2, 32.3) |
| Masahet Island | Bulamue | 275 | 37 | 5.4 (-1.9, 12.7) |
|  | Malal/Dot | 317 | 41 | 14.6 (3.8, 25.5) |
|  | Matatokuen | 322 | 40 | 15.0 (3.9, 26.1) |
|  | Ton | 110 | 97 | 0.1 (-0.01, 0.03) |
|  | Mosoi | 765 | 27 | 22.2 (6.5, 37.9) |
| Mahur Island | Kuelam | 335 | 51 | 13.7 (4.3, 23.2) |
|  | Lakamelem | 324 | 50 | 4.0 (-1.4, 9.4) |
|  | Lih | 248 | 39 | 15.4 (4.1, 26.7) |
