# Supplementary material for: Human and entomological determinants of malaria transmission in the Lihir Islands of Papua New Guinea: A cross-sectional study
Source: PLoS Negl Trop Dis. 2025 Jan 3;19(1):e0012277. doi: 10.1371/journal.pntd.0012277 (PMC11734946; doi:10.1371/journal.pntd.0012277)
Supplement: S3 Table — Abbreviations: CI (Confidence Interval), IR (Incidence Risk), IRR (Incidence Risk Ratio), MIZ (Mine-impacted zone). Binomial regression model was used to estimate IRR with 95% CI and p-values. (DOCX) [file pntd.0012277.s006.docx]

| **Variable** | | **Incidence/1000 inhabitants** | **IR/1000 inhabitants (95% CI)** | **IRR (95% CI)** | **p-value** |
| --- | --- | --- | --- | --- | --- |
| Age (years) | < 4 | 780 | 777.6 (760.4 to 794.2) | Reference | Reference |
|  | ≥ 4 to < 9 | 440 | 435.6 (420.8 to 450.4) | <0.001 | 0.56 (0.54 to 0.58) |
|  | ≥ 9 to < 15 | 450 | 460.0 (440.7 to 479.4) | <0.001 | 0.59 (0.56 to 0.62) |
|  | ≥ 15 | 250 | 249.0 (242.6 to 255.6) | <0.001 | 0.32 (0.31 to 0.33) |
| Geographic area | Aniolam MIZ | 142 | 141.7 (135.8 to 147.7) | Reference | Reference |
|  | Aniolam non-MIZ | 596 | 596.4 (586.5 to 606.2) | <0.001 | 4.21 (4.03 to 4.4) |
|  | Malie Island | 828 | 827.9 (799.8 to 853.5) | <0.001 | 5.84 (5.54 to 6.15) |
|  | Masahet Island | 116 | 116.3 (101.6 to 132.2) | 0.004 | 0.82 (0.71 to 0.94) |
|  | Mahur Island | 712 | 711.8 (682.0 to 740.2) | <0.001 | 5.02 (4.74 to 5.32) |
