# Supplementary material for: Human and entomological determinants of malaria transmission in the Lihir Islands of Papua New Guinea: A cross-sectional study
Source: PLoS Negl Trop Dis. 2025 Jan 3;19(1):e0012277. doi: 10.1371/journal.pntd.0012277 (PMC11734946; doi:10.1371/journal.pntd.0012277)
Supplement: S4 Table — Abbreviations: CI (Confidence Interval), N (number), Prop (proportion). (DOCX) [file pntd.0012277.s007.docx]

| **Habitat type** | **Surveyed habitats**  **(N)** | ***An. farauti***  **Prop (95% CI)** | ***An. punctulatus***  **Prop (95% CI)** |
| --- | --- | --- | --- |
| Artificial containers | 470 | 0.015 (0.006-0.030) | 0.002 (0.0001-0.012) |
| Coconut shells | 45 | 0.000 | 0.000 |
| Pig wallows | 45 | 0.022 (0.001-0.118) | 0.022 (0.001-0.118) |
| Drainage channels | 29 | 0.103 (0.029-0.242) | 0.051 (0.006-0.173) |
| Forest swamps | 13 | 0.154 (0.019-0.455) | 0.000 |
| Transient puddles | 12 | 0.167 (0.021-0.484) | 0.000 |
| Rivers | 11 | 0.000 | 0.000 |
| Drainages | 10 | 0.000 | 0.000 |
| Permanent ground water | 8 | 0.25 (0.032-0.651) | 0.000 |
| Tree holes | 4 | 0.000 | 0.000 |
| Coastal streams | 3 | 0.000 | 0.000 |
| Others | 70 | 0.186 (0.103-0.297) | 0.000 |
| TOTAL | 784 | 0.046 (0.032.-0.063) | 0.024 (0.015-0.038) |
