# Supplementary material for: Human and entomological determinants of malaria transmission in the Lihir Islands of Papua New Guinea: A cross-sectional study
Source: PLoS Negl Trop Dis. 2025 Jan 3;19(1):e0012277. doi: 10.1371/journal.pntd.0012277 (PMC11734946; doi:10.1371/journal.pntd.0012277)
Supplement: S1 File — (DOCX) [file pntd.0012277.s008.docx]

**SUPPLEMENTARY METHODS**

**Light microscopy detection of *Plasmodium* *spp.* parasites for assessing prevalence**

Collected blood films were fixed and stained for 30 minutes with Giemsa 4% at the local laboratory upon arrival from the field, and they were later sent to the PNG Institute of Medical Research (IMR) Vector-borne Diseases Unit at Madang (PNG) for examination. The slides were examined at 1000x magnification by two independent level 1-2 microscopists having completed WHO quality assurance courses. A sample was considered negative after examining one hundred fields of view. When a parasite was observed, counts of white cells and parasites were conducted until 200 white cells had been counted, or until 500 if there were less than 10 parasites/200 leukocytes. The parasite count was calculated assuming a white cell count of 8,000 cells/ μL. Gametocyte stages were recorded separately. If the first two reads were discrepant beyond the required tolerance, a different WHO-certified level 1 microscopist re-examined the slides.

**DNA extraction and molecular detection of *Plasmodium* *spp* parasites for assessing prevalence**

Filter papers with blood spots were dried in the field, placed in separate zip-lock bags, and stored at -20 ºC. Subsequently, they were sent to the IMR Vector-borne Diseases Unit in Madang (PNG) for further processing. DNA was extracted using FavorPrep 96-well Genomic DNA kit (FAVORGEN®) and performed according to the manufacturer protocol for extraction of genomic DNA from blood. Following DNA extraction, a generic quantitative PCR (QMAL) that amplifies a conserved region of the 18S rRNA gene was run on all samples (1); and for all positive samples, a species-specific quantitative PCRs (qPCR) detecting all *Plasmodium* species were performed as previously described (2). Finally, in PCR QMAL positive samples that yielded negative by the species-specific qPCR, ultra-sensitive qPCRs targeting Pf-varATS for *P. falciparum* and Pv-mtCOX1 for *P. vivax* were conducted (3, 4).

**Procedures for the human landing catches and analysis of adult mosquitoes**

Participants were seated comfortably with the lower part of their legs (from the knee to the feet) exposed to host-seeking mosquitoes. Mosquitoes that rested and attempted to bite on the exposed part of the legs were captured using a mouth aspirator aided by a flashlight/torch to see the mosquitoes (5) and stored in a paper cup with a screened lid classifying the date and hour of the night, and location (geolocation, household, indoor/outdoor). The human landing catches (HLC) were done hourly from 6:00 pm to 6:00 am each night during a collection round, simultaneously conducted next to sleeping spaces inside the houses (indoor collections) and outside but near the houses (outdoor collections). A total of 4 nights of HLC collection round was conducted in each village across 10 households, except in 1 village in Aniolam MIZ where only 7 households participated). Mosquitoes were separated into their respective genera with the aid of a light microscope. Female *Anopheles* were morphologically identified to species (6), and placed in a 2-mL microcentrifuge tube packed with silica desiccant with a unique identification number. This allowed for analyses of the mosquito biting frequency, biting time, indoor vs. outdoor biting, and the entomological inoculation rate (EIR).

**Molecular identification of *Anopheles*species and sporozoites**

At the entomology unit at PNGIMR Vector-borne diseases unit (Madang, PNG), the abdomen of each *Anopheles* mosquito was separated from the rest of the body and DNA was extracted from the abdomen-detached body part (i.e., head and prothorax) using DNeasy Blood and Tissue Kit (Product number: 69582; Qiagen, Valencia, CA, USA) and later FavorPrep® DNA extraction kits (Favorgen Biotech Corp, Ping Tung, Taiwan). Samples were analysed using a standard PCR method by Internal Transcribed Spacer 2 (ITS2) region restriction fragment length polymorphism (RFLP) for species determination (7). Sporozoite positive mosquitoes were Identified using a multiplex quantitative PCR with two fluorescent-labelled TaqMan probes targeting the 18S rRNA gene of *P. falciparum* and *P. vivax* as previously described (8, 9). The data on PCR-positive mosquitoes was used to estimate the EIR (10).

**REFERENCES**

1. Wampfler R, Mwingira F, Javati S, Robinson L, Betuela I, Siba P, et al. Strategies for detection of Plasmodium species gametocytes. PLoS One. 2013;8(9):e76316.

2. Rosanas-Urgell A, Mueller D, Betuela I, Barnadas C, Iga J, Zimmerman PA, et al. Comparison of diagnostic methods for the detection and quantification of the four sympatric Plasmodium species in field samples from Papua New Guinea. Malar J. 2010;9:361.

3. Hofmann N, Mwingira F, Shekalaghe S, Robinson LJ, Mueller I, Felger I. Ultra-sensitive detection of Plasmodium falciparum by amplification of multi-copy subtelomeric targets. PLoS Med. 2015;12(3):e1001788.

4. Gruenberg M, Moniz CA, Hofmann NE, Wampfler R, Koepfli C, Mueller I, et al. Plasmodium vivax molecular diagnostics in community surveys: pitfalls and solutions. Malar J. 2018;17(1):55.

5. World Health Organization. Manual on practical entomology in malaria prepared by the WHO Division of Malaria and Other Parasitic Diseaases. Geneva1995.

6. Belkin J. The Mosquitoes of the South Pacific (Diptera, Culicidae), Vol. 2: Cambridge University Press; 1962.

7. Beebe NW, Saul A. Discrimination of all members of the Anopheles punctulatus complex by polymerase chain reaction--restriction fragment length polymorphism analysis. The American journal of tropical medicine and hygiene. 1995;53(5):478-81.

8. Keven JB, Artzberger G, Gillies ML, Mbewe RB, Walker ED. Probe-based multiplex qPCR identifies blood-meal hosts in Anopheles mosquitoes from Papua New Guinea. Parasit Vectors. 2020;13(1):111.

9. Kamau E, Alemayehu S, Feghali KC, Saunders D, Ockenhouse CF. Multiplex qPCR for detection and absolute quantification of malaria. PLoS One. 2013;8(8):e71539.

10. Smith RC, Jacobs-Lorena M. Plasmodium-Mosquito Interactions: A Tale of Roadblocks and Detours. Adv In Insect Phys. 2010;39:119-49.
